# Low-temperature direct bonding of InP and diamond substrates under atmospheric conditions

**DOI:** 10.1038/s41598-021-90634-4

**Published:** 2021-05-27

**Authors:** Takashi Matsumae, Ryo Takigawa, Yuichi Kurashima, Hideki Takagi, Eiji Higurashi

**Affiliations:** 1 Device Research Technology Institute, National Institute of Advanced Industrial Science and Technology, Ibaraki, 305-8564 Japan; 2 Graduate School of Information Science and Electrical Engineering, Kyushu University, 744 Motooka, Nishi-ku, Fukuoka, 819-0395 Japan

**Keywords:** Electrical and electronic engineering, Surface chemistry, Materials for devices, Materials science, Nanoscience and technology

## Abstract

An InP substrate was directly bonded on a diamond heat spreader for efficient heat dissipation. The InP surface activated by oxygen plasma and the diamond surface cleaned with an NH_3_/H_2_O_2_ mixture were contacted under atmospheric conditions. Subsequently, the InP/diamond specimen was annealed at 250 °C to form direct bonding. The InP and diamond substrates formed atomic bonds with a shear strength of 9.3 MPa through an amorphous intermediate layer with a thickness of 3 nm. As advanced thermal management can be provided by typical surface cleaning processes followed by low-temperature annealing, the proposed bonding method would facilitate next-generation InP devices, such as transistors for high-frequency and high-power operations.

## Introduction

The electronics industry has utilized indium phosphide (InP) to engineer advanced components. Because InP has high electron velocities, low contact resistances, and large heterojunction offsets in the InGaAs/InP system, InP-based electronic devices have been used in high-frequency applications with a maximum oscillation frequency of over 1 THz^1–4^. In addition, communications and networking specialists have been working on THz monolithic integrated circuits (TMIC) with InP high electron mobility transistors (HEMTs) and heterojunction bipolar transistors (HBTs)^5–7^. The direct bandgap of InP is useful in photonic device applications, such as InP laser/modulator/photodetector systems for next-generation optical communications^8–11^. Accompanying the demand for miniaturization and high-power operation^12–17^, the power density of these devices has drastically increased. Consequently, InP-based electronic devices suffer from heat dissipation problems due to the low thermal conductivity of 68 W/m/K (Si: 130 W/m/K)^18–20^.

For efficient heat dissipation, semiconductor researchers have developed an integration technique for devices on a diamond heat spreader, which has the highest thermal conductivity amongst solid materials (2200 W/m/K). For example, Ksenia Nosaeva et al. transferred a diamond heat-spreading layer on the InP HBT embedded in benzocyclobutene (BCB) resin^21^. Andreas Beling et al. integrated InP photodiodes on a diamond sub-mount by flip-chip bonding using metal bonding layers^22,23^. Direct bonding of the device and diamond is ideal to mitigate thermal resistance because the thermal conductivities of these bonding materials are an order of magnitude smaller than that of diamond. In particular, there have been intensive studies regarding the direct and indirect bonding of Ga-based materials (i.e., GaN^24–26^, GaAs^27–30^, and InGaP^31^) onto diamond substrates. However, studies on the direct bonding of InP and diamond substrates are scarce.

**Figure 1:**
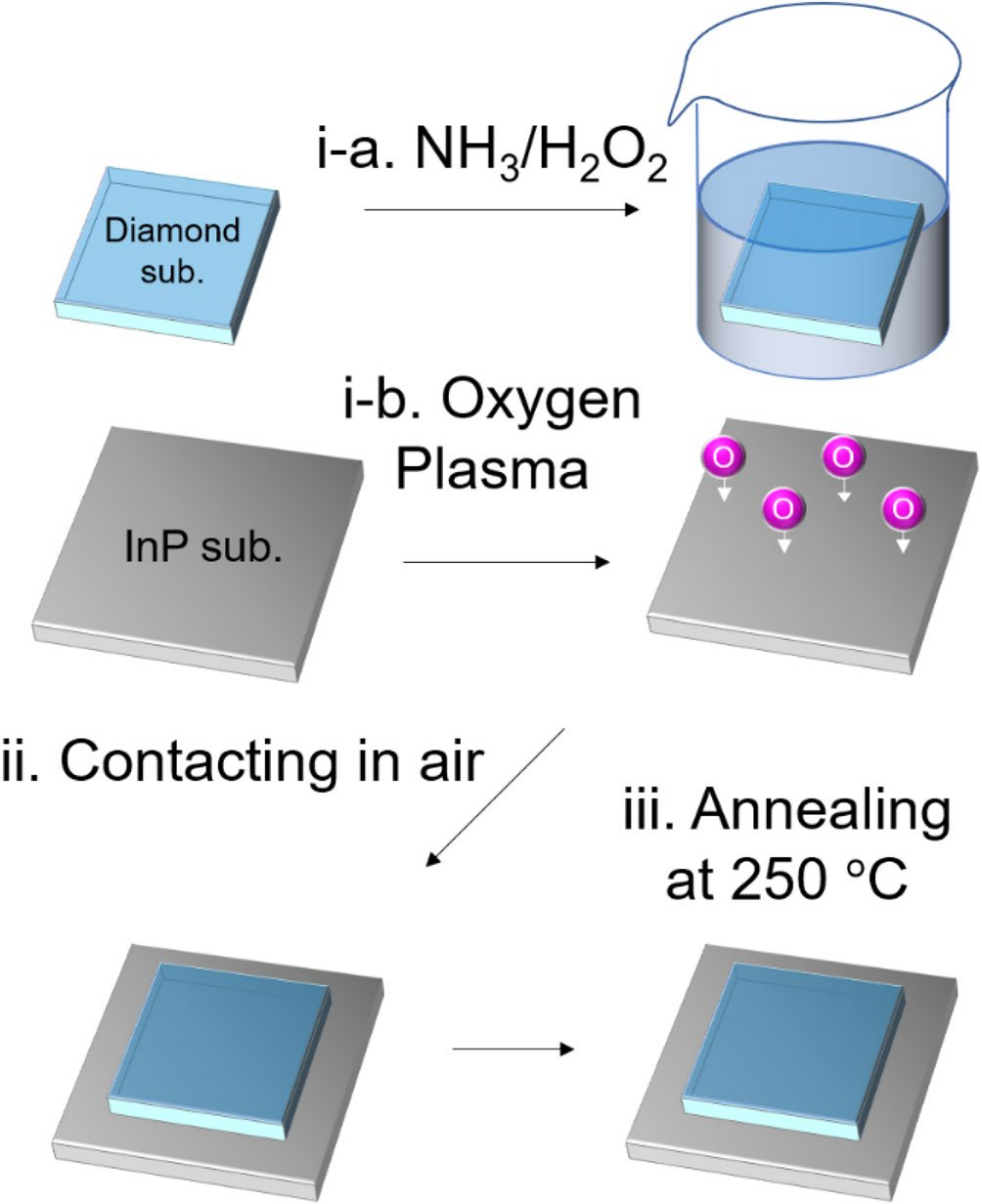
Experimental procedure to directly bond the InP and diamond substrates at low temperatures under atmospheric conditions.

Our research group developed and reported a direct bonding method for semiconductor substrates (i.e., Si, Ga_2_O_3_) on a diamond heat spreader^32–36^. We found that OH groups were formed on a diamond surface treated with oxidizing solutions, such as H_2_SO_4_/H_2_O_2_^32^ and NH_3_/H_2_O_2_ mixtures. Moreover, the OH-terminated diamond surface forms direct bonding with the OH-terminated semiconductor substrate by thermal dehydration at approximately 200 °C. The semiconductor substrates are typically OH-terminated using plasma activation^37^. While studies on the bonding of InP and diamond are scarce, optoelectronics scientists have developed multifunctional devices using direct bonding techniues^38–40^ and achieved the direct bonding of oxygen-plasma-activated InP lasers and Si waveguides^41–44^. Consequently, the InP surface activated by the oxygen plasma can be directly bonded with the OH-terminated diamond surface. To test this hypothesis, we proposed direct bonding of InP and diamond substrates and investigated nanostructures of the InP/diamond bonding interface, as illustrated in Fig. 1.

## Results

**Figure 2:**
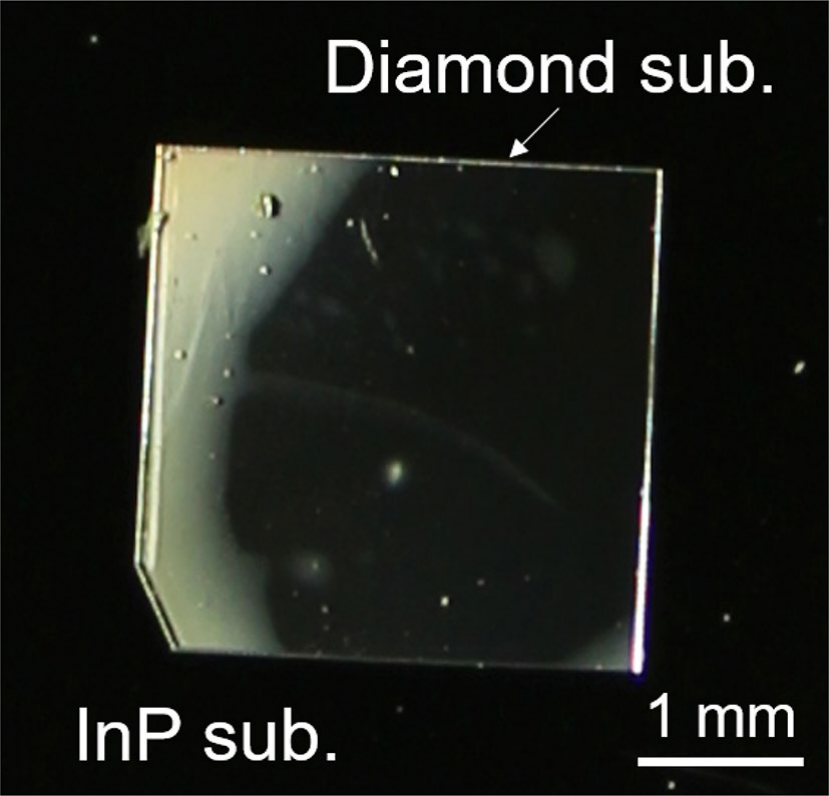
Diamond substrate bonded on the InP substrate. Bright areas are due to unbonded areas at InP/diamond interface.

Figure 2 shows the diamond substrate bonded on the surface of the InP substrate. The bonding interface can be observed through the transparent diamond substrate. Diffused reflection due to the gaps between the substrates was observed where the surfaces were not bonded. While there were some bright spots, Fig. 2 indicates that three-quarters of the contacted area was successfully bonded. Voids with diameters of approximately 0.1 mm were formed due to particles on the substrate surface. The large unbonded regions at the corners of diamond substrates resulted from the convex diamond surface (see the supplement of^34^). If the environmental cleanliness and substrate flatness are improved, direct bonding will be formed at most of the contacted area. When a shear force of 9.3 MPa (84 N for 3 × 3 mm) was applied to the bonded diamond substrate, fracture at the bonding interface and cleavage along the InP (110) face were observed. In our previous studies, the bonding strength of Si/diamond^36^ and Ga_2_O_3_/diamond^34^ was fractured by the shear strength of 31.8 and 14 MPa, respectively. While the bonding interface of InP/diamond was low compared with them, the strength satisfies the die shear strength of MIL STD 883E.

**Figure 3:**
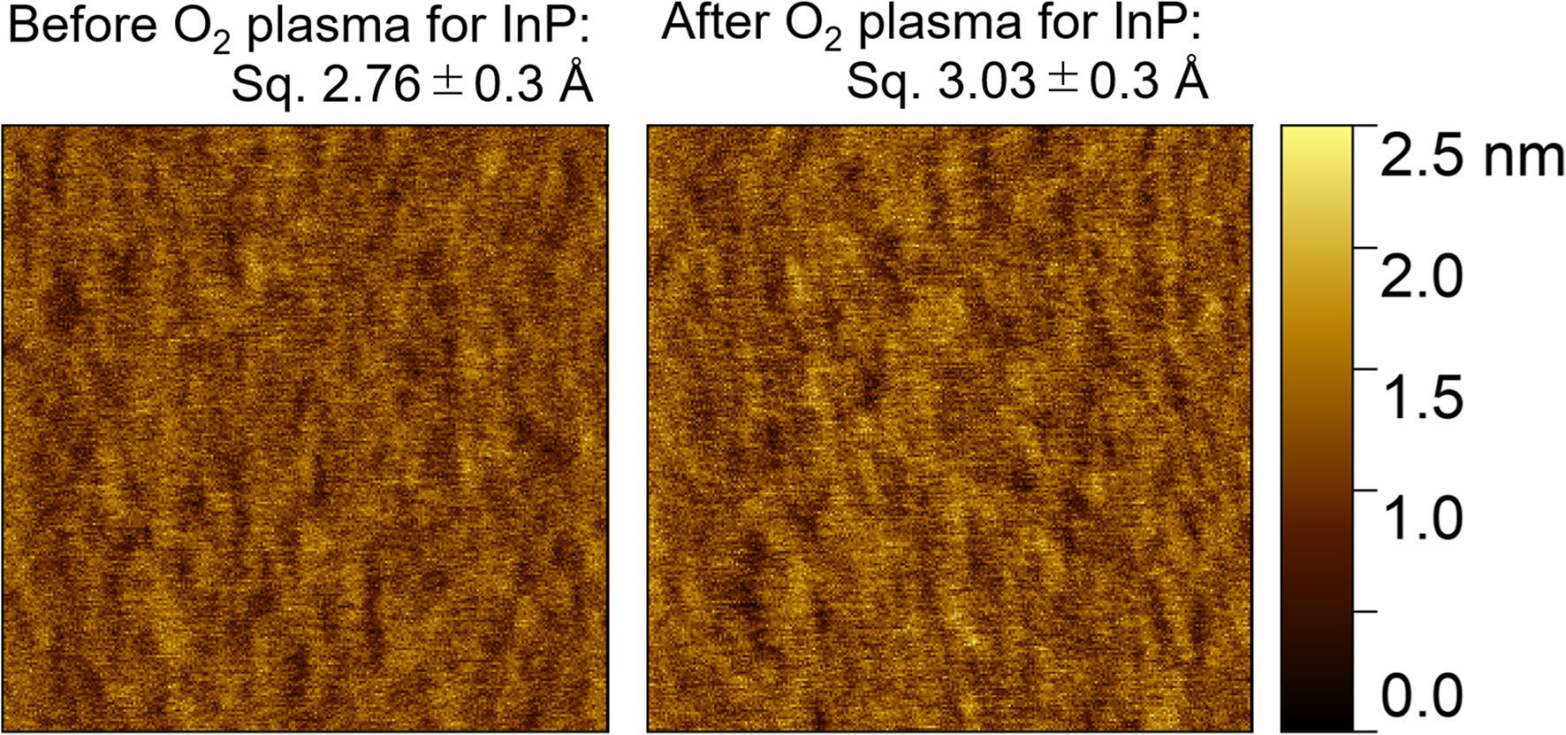
AFM surface image of InP substrate before and after the oxygen plasma irradiation. The activated surface was sufficiently smooth for bonding formation.

Surfaces are required to be sufficiently smooth for direct bonding; the root mean square (RMS) roughness is preferably less than ~ 5 Å^45^. The diamond substrate used in this study had an atomically smooth surface with an RMS roughness of less than 3 Å, which was reported in our previous study^36^. The InP substrate surface was investigated using an atomic force microscope (AFM), as shown in Fig. 3. The RMS roughness of the InP substrate surface was initially 2.76 ± 0.3 Å. Thereafter, the surface roughness after the oxygen plasma irradiation was similar as the RMS roughness was 3.03 ± 0.3 Å; it was sufficiently smooth for bonding formation.

**Figure 4:**
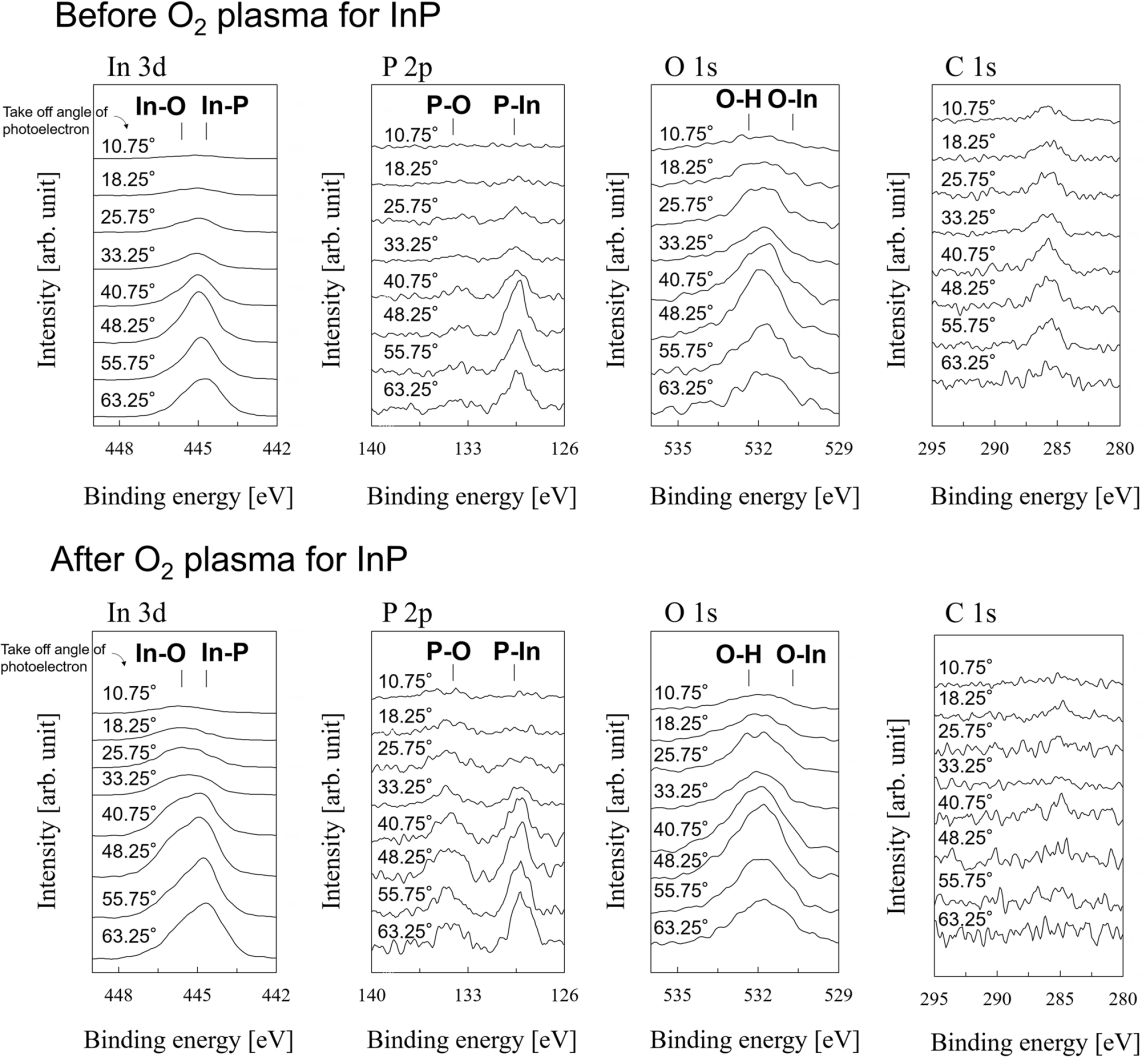
XPS spectra of InP substrate before and after oxygen plasma irradiation. The activated InP surface was functionalized with OH groups.

The surface chemical composition of the InP substrate was investigated through angle-resolved X-ray photoelectron spectroscopy (XPS), as depicted in Fig. 4. The measurement depth depended on the take-off angle of the photoelectrons; the inelastic mean free path (IMFP) was calculated at approximately 1 and 4 nm for angles of 10.75° and 63.25°, respectively. Before plasma irradiation, the amounts of In–O and P–O bonds were relatively small, and organic contaminants were present on the surface. This indicated that the OH groups detected at the surface probably resulted from C–OH bonds, owing to contaminants. However, organic contaminants rarely existed, and In–O and P–O bonds were present on the plasma-activated InP surface. Thus, the OH bonds detected on the surface were possibly attributed to the In–OH, P–OH, or both groups generated on the InP substrate. Our previous study suggested that the diamond substrate cleaned with the NH_3_/H_2_O_2_ mixture was terminated with the C–OH groups^36^. Consequently, the OH groups on the InP and diamond substrates probably reacted with each other during the bonding process.

**Figure 5:**
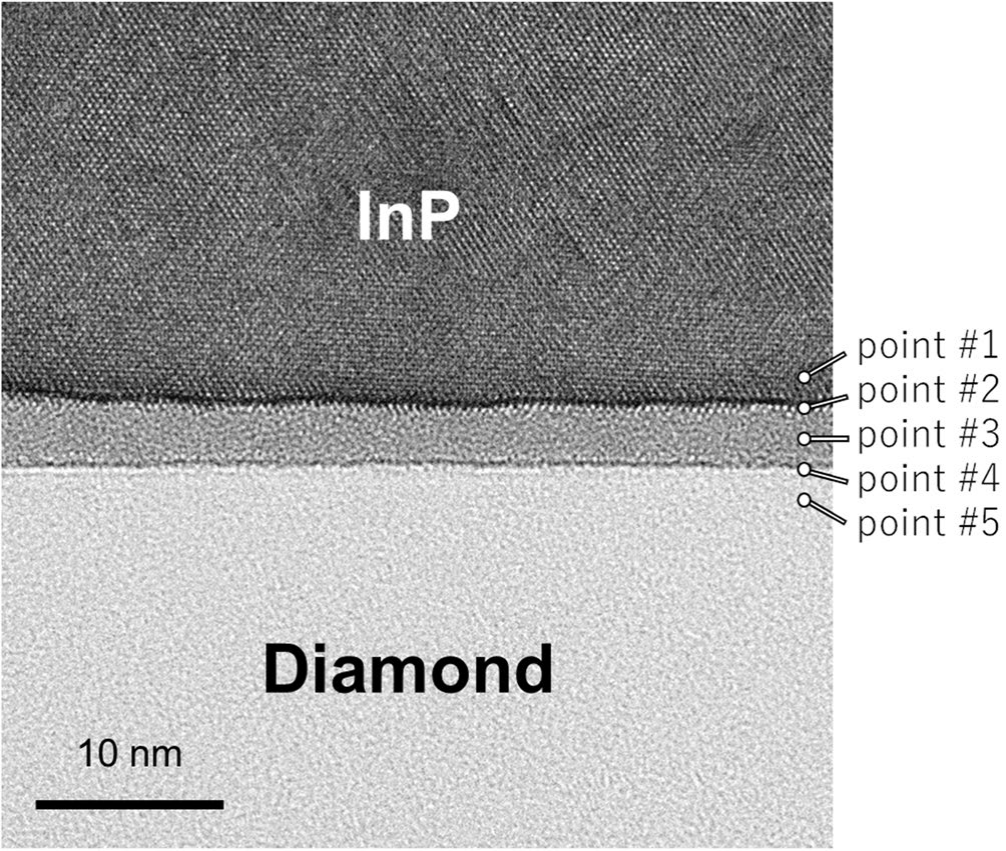
Cross-sectional TEM image of the InP/diamond bonding interface. The electron beam was set to emphasize the crystallinity of InP.

**Figure 6:**
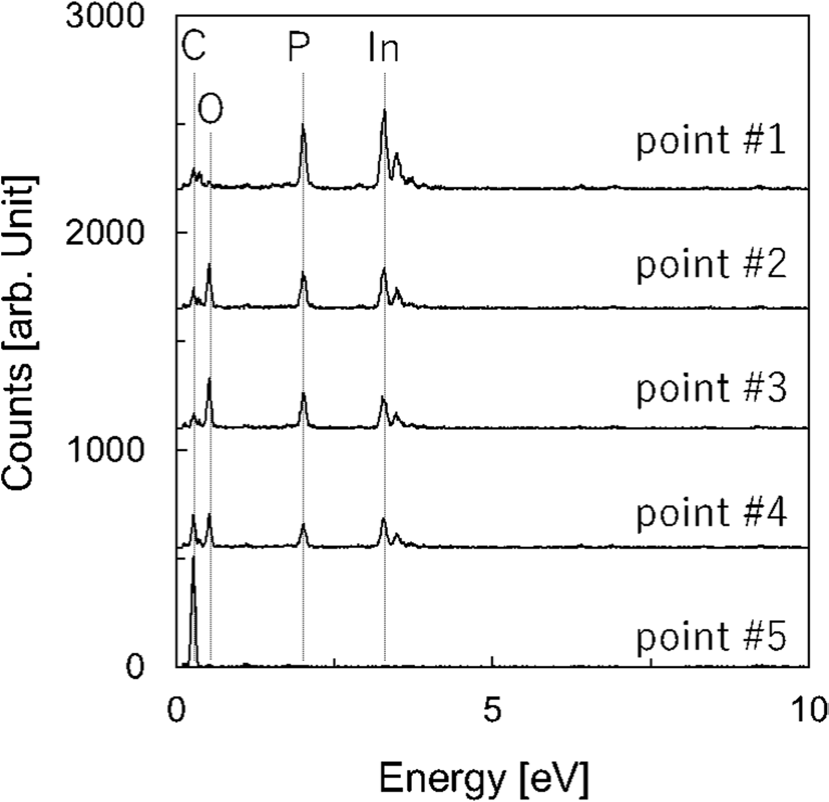
EDX spectra acquired at different points of the bulk and the bonding interface.

The nanostructure of the InP/diamond bonding interface was observed using a transmission electron microscope (TEM), as shown in Fig. 5. For the observation, the thickness of the InP substrate, bonded with diamond, was reduced to 10 µm by grinding. Subsequently, the ultra-thin TEM specimen was prepared using a focused ion beam (FIB). The incident angle of the electron beam was set parallel to the InP < 110 > direction. As shown in Fig. 5, the InP and diamond substrates formed atomic bonds without cracks or nanovoids. Moreover, an amorphous layer with a thickness of approximately 3 nm was observed at the bonding interface. Figure 6 depicted the compositional analysis obtained using energy-dispersive X-ray spectroscopy (EDX). The amorphous layer at the bonding interface is composed of In, P, O, and C. It is known that the InP/Si interface bonded using oxygen plasma is composed of In, P, and O^44^. The C atoms supposedly diffused into the oxide layer on the InP substrate formed by the oxygen plasma; the formation of the intermediate oxide layer is unavoidable in the case of the bonding under atmospheric conditions. It was assumed that the thermal conductivity of the intermediate layer was low but significantly thin compared with conventional approaches (e.g. 2–4-µm-thick metal layers^21,22^). Thus, it was supposed that the InP/diamond bonding technique would contribute to efficient heat dissipation from the InP electronic devices.

## Conclusions

In this study, we demonstrated the direct bonding of InP and diamond substrates to improve the heat dissipation of InP-based electronic devices. The InP substrate activated by oxygen plasma was contacted with the diamond substrate that was cleaned with a mixture of NH_3_, H_2_O_2_, and H_2_O under atmospheric conditions. Direct bonding was formed by annealing the contacted specimen at 250 °C. As both surfaces were atomically smooth after the pre-bonding treatments, the InP and diamond substrates successfully generated direct bonding with a shear strength of 9.3 MPa. The interfacial analysis revealed that they were bonded through an amorphous intermediate layer with a thickness of approximately 3 nm without cracks or nanovoids. The oxygen plasma treatment and cleaning with the NH_3_, H_2_O_2_, and H_2_O mixtures in the pre-bonding step are commonly applied substrate cleaning processes in the electronics industry. The subsequent bonding step can be realized using low-temperature annealing under atmospheric conditions. Because advanced thermal management can be achieved by simple procedures, this bonding technique would contribute to future InP devices with higher integration and power densities.

## Method

In this study, commercially available InP and diamond substrates were directly bonded as received conditions, as illustrated in Fig. 1. Three-square-millimeter diamond (111) substrates with a thickness of 300 μm (from EDP Corp.) were bonded on a three-inch-diameter InP (100) wafer with a thickness of 500 μm (from Sumitomo Electric Industries, Ltd).

The diamond substrates were cleaned with a mixture of 10 mL of NH_3_ solution (28%), 10 mL of H_2_O_2_ solution (35%), and 50 mL of deionized water at 75 °C for 10 min. The diamond substrates were rinsed in deionized water and blown by nitrogen gas for drying. The InP substrates were activated with reactive ion etching equipment (QAP-1000, Bondtech). The plasma at a power of 200 W irradiated the InP surface for 30 s under an O_2_ pressure of 60 Pa and an O_2_ mass flow rate of 20 mL/min. In the contacting step, the activated InP substrate was placed on a Peltier cooler at 14 °C for approximately 30 s in our clean room (temperature: 23 °C, relative humidity: 40%), and then the diamond substrate was placed on the InP substrate. The cooling process developed condensed water molecules that are believed to promote hydrogen bond networks between the InP and diamond substrates. The contacted specimen was annealed at 250 °C for 24 h under a load of approximately 1 MPa.

The bonding quality was evaluated using a shear tester (4000Plus, Nordson DAGE). The surface roughness of the InP substrate was investigated using the AFM (L-trace, Hitachi). The surface chemical composition was studied using XPS (VG Theta Probe, Thermo Fisher Scientific). The nanostructure of the InP/diamond bonding interface was investigated using the TEM and EDX (JEM-ARM200F, JEOL).
